# Osteomyelitis in the Nonunion of a Patellar Fracture

**DOI:** 10.7759/cureus.67547

**Published:** 2024-08-22

**Authors:** Athena Z Bennani, Brian Chegwidden, Liam Jones, Constantino G Lambroussis, Lorrie Penfield

**Affiliations:** 1 College of Medicine, Lake Erie College of Osteopathic Medicine, Elmira, USA; 2 Osteopathic Medicine/Family Medicine, Lake Erie College of Osteopathic Medicine, Elmira, USA; 3 Internal Medicine/Medical Education, Arnot Ogden Medical Center/Lake Erie College of Osteopathic Medicine, Elmira, USA

**Keywords:** non-hematogenous, hematogenous, nonunion, osteomyelitis, fracture, patellar, patella

## Abstract

Osteomyelitis is by definition any infection of the bone. It can have a hematogenous or non-hematogenous mechanism of infection, but comorbidities such as cardiovascular disease, diabetes mellitus, and the presence of orthopedic hardware can increase the risk of osteomyelitis. Our case focuses on a 64-year-old Caucasian female with multiple comorbidities who presented with a fractured right patella that was not healing four months after the occurrence of the fracture. The patient reported cramping, fasciculations, and severe pain that was worsening. She also reported that she received nine X-rays from different orthopedists before receiving an MRI, ordered by internal medicine. The MRI showed a small knee effusion with mild generalized edema of the nearby subcutaneous tissues and evidence of nonunion of the fracture as evidenced by fracture fragments of the right patella. The MRI additionally showed increased signal in the bone fragments of the right patella, as well as the anterior and posterior superior rims of the right tibial plateau, concerning for potential osteomyelitis. Referral to infectious disease confirmed the diagnosis of patellar osteomyelitis, a rather rare diagnosis. The patient was promptly started on cefdinir and doxycycline, and within days of starting antibiotic therapy, her right knee pain was reduced to zero. Surgical debridement was not necessary, and the patient was able to resume her daily activities with the pain resolved. The possibility of patients only having to undergo antibiotic treatment for patellar osteomyelitis improves their chances of a full recovery and reduces the risks associated with undergoing surgical debridement.

## Introduction

By definition, osteomyelitis is any infection of the bone, with approximately 50,000 cases annually in the United States. It is categorized into non-hematogenous and hematogenous diseases. Non-hematogenously infected bone occurs due to the direct inoculation or direct spread of infection from the adjacent tissue into the bone. Some common places of non-hematogenous bacterial seeding are long bones, especially in adults [1]. However, a particularly rare place for osteomyelitis to occur is the patella. Since 1829, there have been less than 100 cases reported [2]. Some factors that could contribute to the development of isolated patellar lesions include trauma, degenerative diseases, primary or metastatic tumors, congenital defects, cysts, and infections [3]. There is very much a lack of cases reported in the literature, without a recommended established standard of care, making each patient encounter a case-by-case basis. 

Two important classification systems for osteomyelitis are the Lew and Waldvogel classification system and the Cierny-Mader classification of osteomyelitis for long bones. According to the Lew and Waldvogel classification system [4], there are two mechanisms in which a bone can be infected non-hematogenously. The first mechanism of infection is non-hematogenous osteomyelitis caused by local spread from an adjacent source of infection, usually caused by trauma, penetrating wounds, surgery, or joint replacement [5,6]. Individuals with foreign body implants are at the highest risk for infection due to factors such as contamination of the implant and/or the wound site [7,8]. The second mechanism of infection is a non-hematogenous osteomyelitis due to vascular insufficiency, occurring mainly in diabetic patients with peripheral neuropathy. This occurs almost always after a soft tissue infection of the foot that eventually spreads to the bone. The combination of hyperglycemia, bone and soft tissue ischemia, and peripheral motor, sensory, and autonomic neuropathy all contribute to an increased risk of infection of an open diabetic foot ulcer [4,9]. 

Lewis and Waldvogel also provide a mechanism of infection for hematogenously infected bone, which results from decreased blood flow velocity within the vascular loops between the metaphysis and the epiphyseal plates, allowing microorganisms to invade the bone [8,9]. Bones are especially vascular during childhood due to normal bone growth and development. Hematogenous osteomyelitis is more commonly seen in male children, especially in highly vascular metaphyseal regions of long bones, such as of the humerus and femur, which are easily damaged by minor trauma [1,10-12]. Hematogenous osteomyelitis is also often found in acute infections in the setting of bacteremia [5,11]. Risk factors include diabetes mellitus, sickle cell disease, endocarditis, the presence of intravascular devices, orthopedic hardware, as well as injection drug use [13,14]. Patellar osteomyelitis is commonly seen in pediatric patients, as the vascularity of the patella peaks around the age of 12, making them susceptible to hematogenous infection [15].

The microbial invasion of bone causes an inflammatory response, which causes an increase in pressure within the medullary bone. The damage within the medullary bone can cause the infection to spread into the cortical bone [7,16]. If left untreated, the infection invades into the periosteum, which compromises the blood flow to the bone, resulting in necrosis and structural instability [7]. Vascular insufficiency further contributes to the progression of infection due to the inability of leukocytes to clear the infection and prevent the healing of the wound [8]. Sequestra, pieces of necrotic bone, are characteristic of a chronic, late-stage osteomyelitis infection [5].

Non-hematogenous osteomyelitis can be either mono- or polymicrobial, while hematogenous osteomyelitis is often monomicrobial [5,7,16]. The most common infecting organism in both hematogenous and non-hematogenous osteomyelitis is *Staphylococcus aureus* since it is part of the natural skin flora [5,9,10,17]. *S. aureus* possesses multiple virulence factors such as adhesins, cytolytic toxins, immunoevasion factors, superantigens, and antioxidant systems, which allows it to colonize and infiltrate wounds [18]. Other organisms that can cause osteomyelitis include coagulase-negative staphylococci, streptococci, and enterococci in all patients [8,16]. *Pseudomonas aeruginosa* and *Serratia marcescens* infections are commonly seen in injection drug users [19]. *Aspergillus* species infections are commonly seen in immunosuppressed patients [20]. Group A and B *Streptococcus pyogenes* and *Kingella kingae *infections are commonly seen in infants and children [10,11,21-23].

Once the mechanism of infection has been established, the Cierny-Mader staging system of osteomyelitis in long bones can be utilized to define the extent of damage [7]. The stages of this system are as follows: In stage 1 or medullary osteomyelitis, the infection exists only in the medullary cavity of the bone. It is associated with acute osteomyelitis [1,16,24]. In stage 2 or superficial osteomyelitis, the infection is isolated to the cortical bone and often results from non-hematogenous osteomyelitis. It is associated with acute osteomyelitis, transitioning into chronic osteomyelitis [1,16,24]. In stage 3 or localized osteomyelitis, the infection involves both cortical and medullary bone; however, it has not caused significant structural instability. It is associated with chronic cases of osteomyelitis [1,16,24]. In stage 4 or diffuse osteomyelitis, there is an invasion of the entire diameter of the bone, accompanied by loss of stability and nonunion of the bone due to the infection. It is also associated with chronic cases of osteomyelitis [1,16,24].

Furthermore, the Cierny-Mader system categorizes the host into three categories: A, B, and C. These categories are as follows: Category A includes relatively normal patients who do not possess any sort of systemic or local compromise that affects the immune surveillance, metabolism, or vasculature of the area of the infection [1,16]. Category B is divided into three parts: Bs, indicating host systemic compromise; Bl, indicating host local compromise; and Bls, indicating both systemic and local compromise within the host. Conditions such as diabetes mellitus, renal impairment, and hepatic impairment contribute to the systemic compromise of the patient, while conditions such as vascular disease, neuropathy, and excessive tobacco use are all contributors to the local compromise of the host [1,16]. Category C patients are so severely compromised that the treatment of their osteomyelitis would cause more harm than benefit [1,16].

Clinicians should be suspicious of an osteomyelitis diagnosis when a patient presents with the following complaints: fever, new or worsening musculoskeletal pain, reduced range of motion of the knee, signs of cellulitis overlying previously implanted orthopedic hardware, traumatic injury including bite and puncture wounds, and diabetes with ulcers that probe to the bone [4,5]. A thorough history of the patient's medical history should clue the clinician to the host's status and allow them to predict the susceptibility and prognosis of the infection. Several other indications can be used to diagnose a patient with osteomyelitis. Inflammatory markers such as erythrocyte sedimentation rate (ESR), C-reactive protein (CRP), and white blood cell (WBC) count clearly indicate infection, immune reaction, and inflammatory processes occurring within the body. Blood cultures revealing causative agents such as *Staphylococcus*, *Streptococcus*, and *Enterococcus* can indicate a hematogenously acquired or an invasive non-hematogenous infection of osteomyelitis [4,16]. 

Radiographic findings such as osteolytic lesions, periosteal reactions, and bone destruction can be seen approximately two weeks after the initial infection [5,18]. MRI is considered the gold standard for diagnosing osteomyelitis due to its ability to demonstrate bone marrow edema, abscesses, and extraosseous disease spread [24]. Contrast CT and nuclear medicine studies are useful alternatives to MRI. Finally, the most definitive diagnostic criteria for osteomyelitis is a bone biopsy with a positive culture [5]. However, this procedure is invasive and is contraindicated for systemically compromised patients who are likely to suffer complications such as blood clots or wound healing failure following this procedure [25]. 

Management of osteomyelitis can be achieved through both medical and surgical treatment. Communication between infectious disease and orthopedic surgery is required in order to create an efficient treatment plan for patients. Most of the common infecting agents of osteomyelitis can be treated with a penicillin, cephalosporin, or macrolide regimen, with alternative treatments also being available for patients who cannot tolerate the use of these [7,16]. Antibiotic treatment typically lasts from four to six weeks. The use of long-term intravenous access catheters for patients with late-stage osteomyelitis is ideal to reduce the length of hospital stays and allow the patient to return to normal daily activities during the recovery process [22]. The Cierny-Mader classification system of osteomyelitis can be utilized to guide treatment. Stage 1 osteomyelitis in both adults and children can be treated solely with antibiotic therapy. Children have a high recovery rate due to the high vascularity of their bones [26]. Stage 2 osteomyelitis should be treated with a superficial debridement and soft tissue coverage followed by a two-week course of antibiotics. Stages 3 and 4 require major debridement of necrotic tissue followed by a 4-6-week regimen of antibiotics to eliminate the infection from the surrounding tissue [24].

## Case presentation

A 64-year-old Caucasian female presents with severe right anterior knee pain. She indicates that the pain has been severe enough that she has been "crying out at night" and unable to sleep well for the past two weeks as the pain has been worsening. She reports that the pain is a 10/10. The pain is described as a burning sensation localized to the right knee; however, the patient also noted cramping as well as fasciculations/spasms of the right knee. She reports having previously fractured her right patella approximately four months ago during a skiing accident. She has been following up with orthopedics since that time. In total, the patient received nine X-rays on her knee over the course of four months following the fracture. Each time, orthopedics stated that her patella was healing and placed her in a brace. 

The patient has a past medical history which includes the following: cerebrovascular accident (CVA) with residual right leg weakness and right foot drop, anxiety, depression, hypertension, hyperlipidemia, coronary artery disease (CAD), atrial fibrillation (on anticoagulation), hypercoagulability, type 2 diabetes mellitus (A1C 7.5%, previous 6.7% 18 months prior), overactive bladder, fractured right patella, fractured right heel, and L5-S1 disc protrusion. She denies any illicit substance use, consumes 3-4 cups of coffee daily, and has not utilized nicotine products or consumed alcohol for the past 10 years. She was a smoker for approximately 35 years, averaging 1-2 packs per day during that time; however, she quit after her CVA 10 years ago. The patient did previously consume alcohol excessively for nearly 40 years; however, she also quit 10 years ago following her CVA. At the present time, our patient is unemployed as she is disabled.

She then was seen by internal medicine, who took note of her worsening knee pain and ordered labs, revealing that the patient potentially had an infection based on her ESR, CRP, A/G ratio, and platelet count. ESR was 128 (reference range 0-30 mm/hr), CRP was 4.8 (reference range 0.0-0.9 MG/DL), A/G ratio from the serum protein electrophoresis was 0.8, and platelets were 481 (reference range 150-450). Internal medicine then determined that an MRI as well as a knee aspiration should be performed to confirm the diagnosis. The knee aspiration was performed; however, zero fluid was able to be aspirated. Non-contrast MRI of the knee was performed and showed a small knee effusion with mild generalized edema of the nearby subcutaneous tissues and no evidence of fusion of the fracture fragments of the right patella consistent with nonunion of the fracture. The MRI additionally showed increased signal in the bone fragments of the right patella, as well as the anterior and posterior superior rims of the right tibial plateau, concerning for potential osteomyelitis. 

The patient was then referred to be seen by an infectious disease specialist. Infectious disease determined that the patient had osteomyelitis and started her on 300 mg cefdinir PO q12hrs and 100 mg doxycycline PO q24hrs, both taken for 28 days. Her pain began to improve three days after initiating the antibiotics prescribed by infectious disease. The patient was seen by rheumatology seven days after starting the antibiotics, and zero pain was noted in the right knee at that time. Synovial fluid was obtained at the rheumatology appointment; however, there was insufficient material obtained for culture. Microscopic examination showed clear fluid, 1-4 WBC/HPF, with no crystals. Rheumatology recommended for the continuation of the antibiotics as prescribed by infectious disease given the patient's significant improvement after their initiation.

## Discussion

Although a rare diagnosis, our patient presents with a case of patellar osteomyelitis following a fracture that occurred four months prior to her visit. Considering her negative blood cultures and knee aspirations, it is likely that our patient had a case of non-hematogenous osteomyelitis. Additionally, the patient's successful recovery using antibiotics alone indicates that the infection was discovered during its acute phase. The mechanism of infection could be either of the two mentioned by Lewis and Waldvogel [4], considering her comorbidities of previous CVA, type 2 diabetes mellitus, CAD, atrial fibrillation, hyperlipidemia, hypercoagulability, hypertension, and a long history of smoking and alcohol use, all of which could lead to vascular insufficiency. According to the Cierny-Mader system, our patient would be placed in the category of Bls, possessing both locally and systemically compromising conditions [16]. Furthermore, the direct seeding of the patellar could have stemmed from any laceration of the skin since the commonly infecting organisms, such as *S. aureus*, are part of the normal skin flora. 

**Figure 1:**
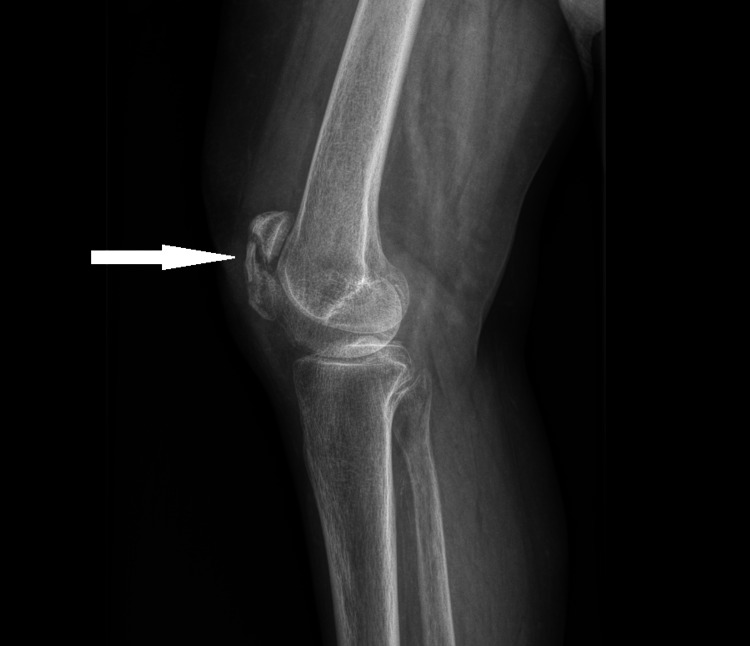
Sagittal view X-ray of the patient's right knee demonstrating a comminuted fracture of the patella, with a 4 mm distraction of the superior pole, overlying soft tissue swelling, and an associated joint effusion.

**Figure 2:**
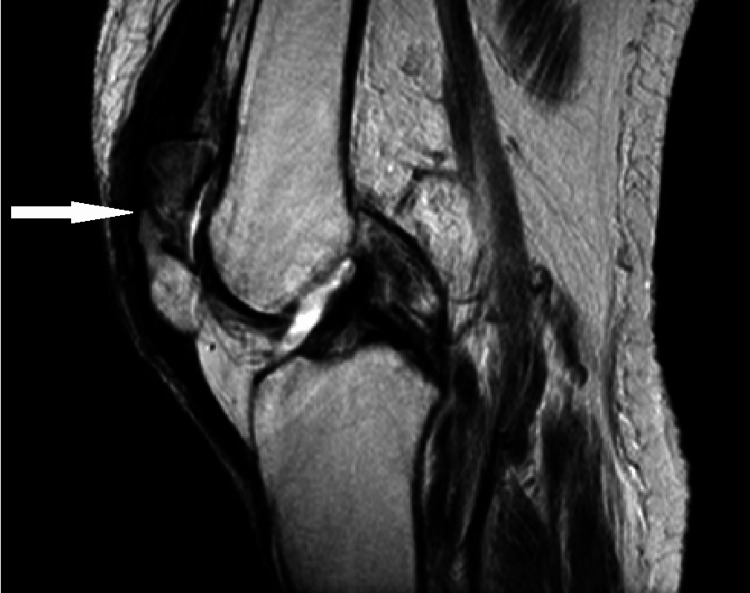
Sagittal view non-contrast T1-weighted MRI of the patient's right knee showing patellar deformity consistent with comminuted fracture shown in Figure 1. No evidence of fusion of the fracture is consistent with nonunion. Small knee effusion with generalized edema of the subcutaneous soft tissues was also seen.

**Figure 3:**
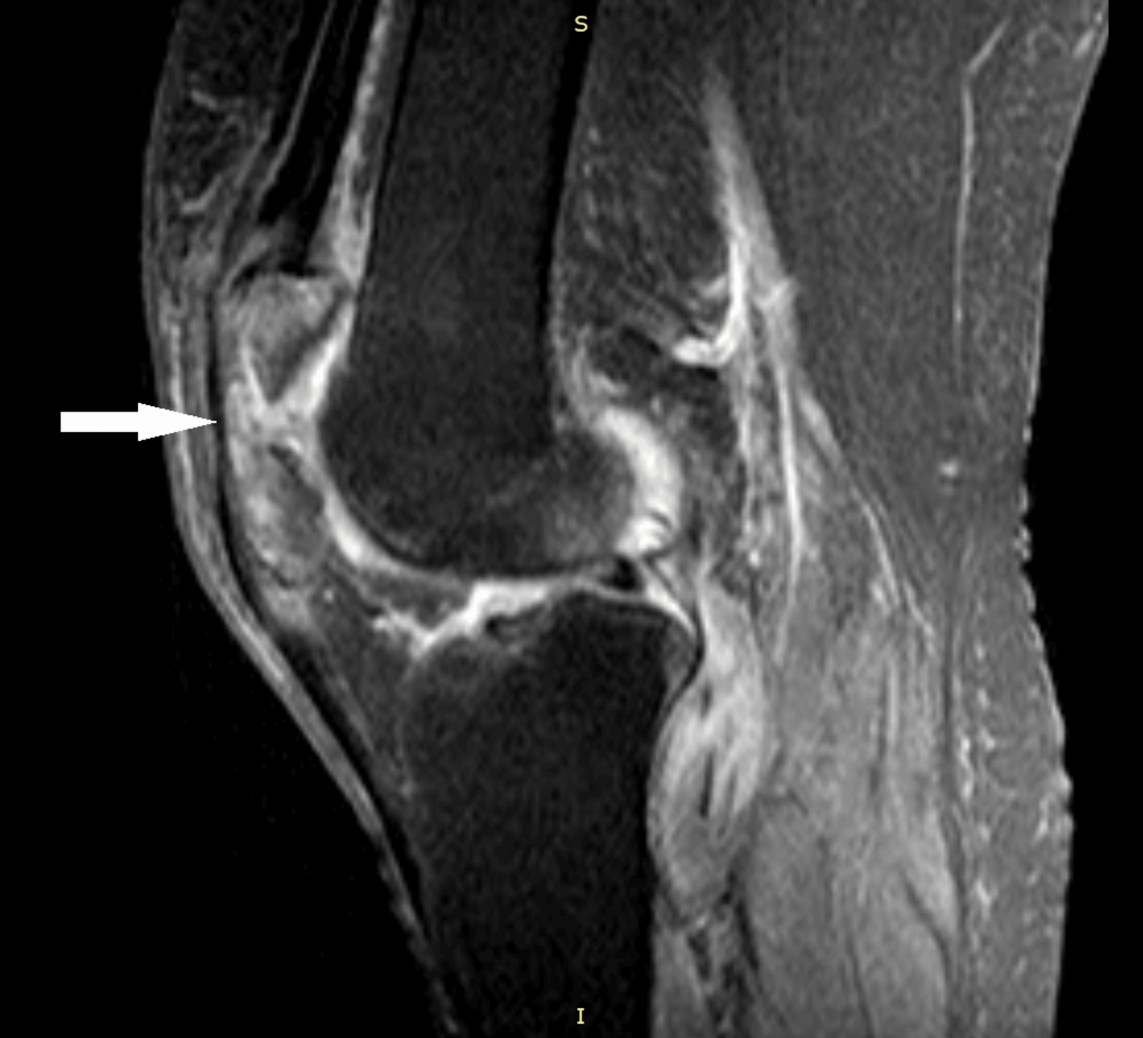
Sagittal view T2-weighted proton density inversion recovery MRI showing increased signal surrounding patellar fragments demonstrating inflammation.

**Figure 4:**
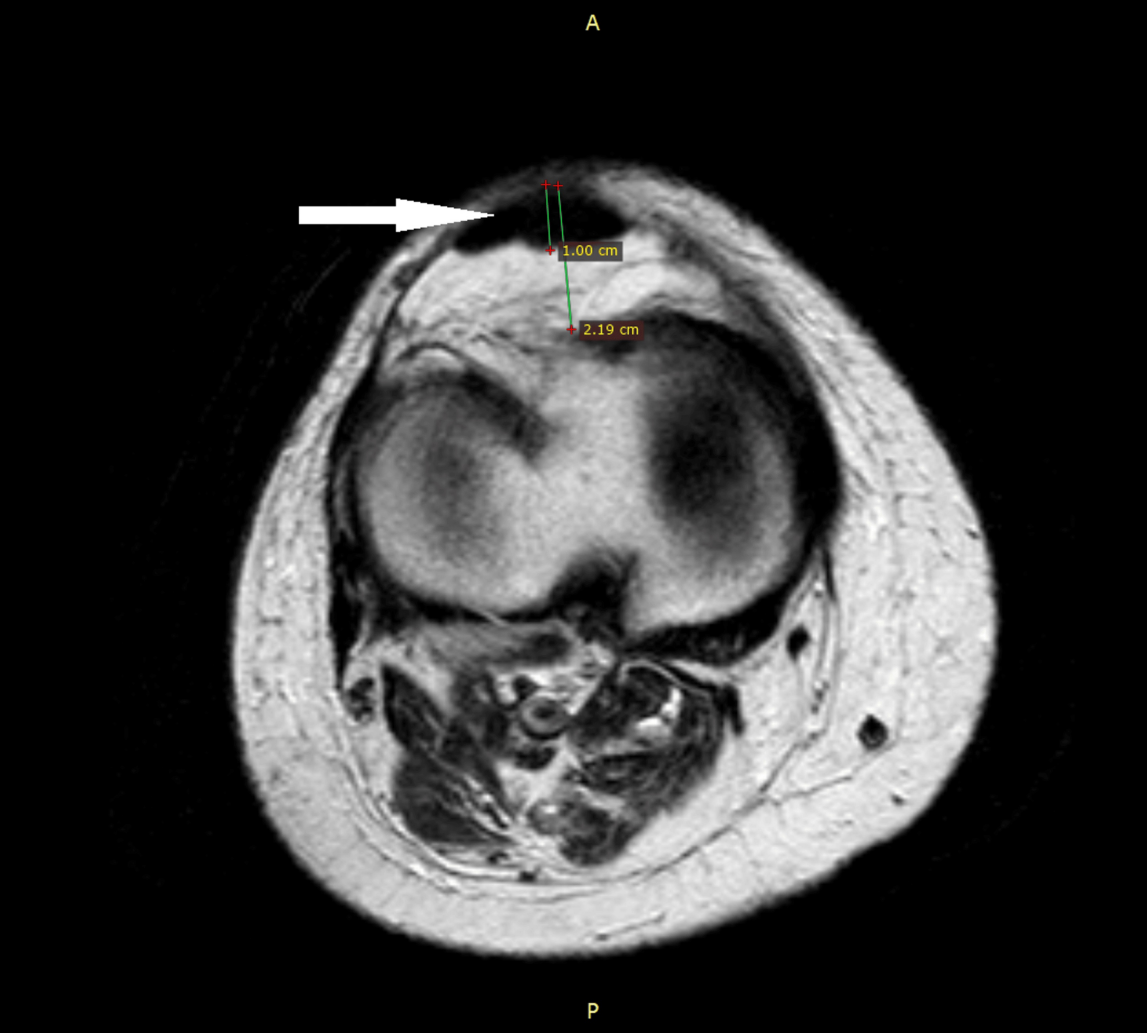
Axial view non-contrast T1-weighted MRI of the patient's right knee demonstrating a large hypodense area involving the inner substance of the patella. The patient's patellar width is 2.19 cm, with a hypodense area encompassing 1 cm of the patellar width.

**Figure 5:**
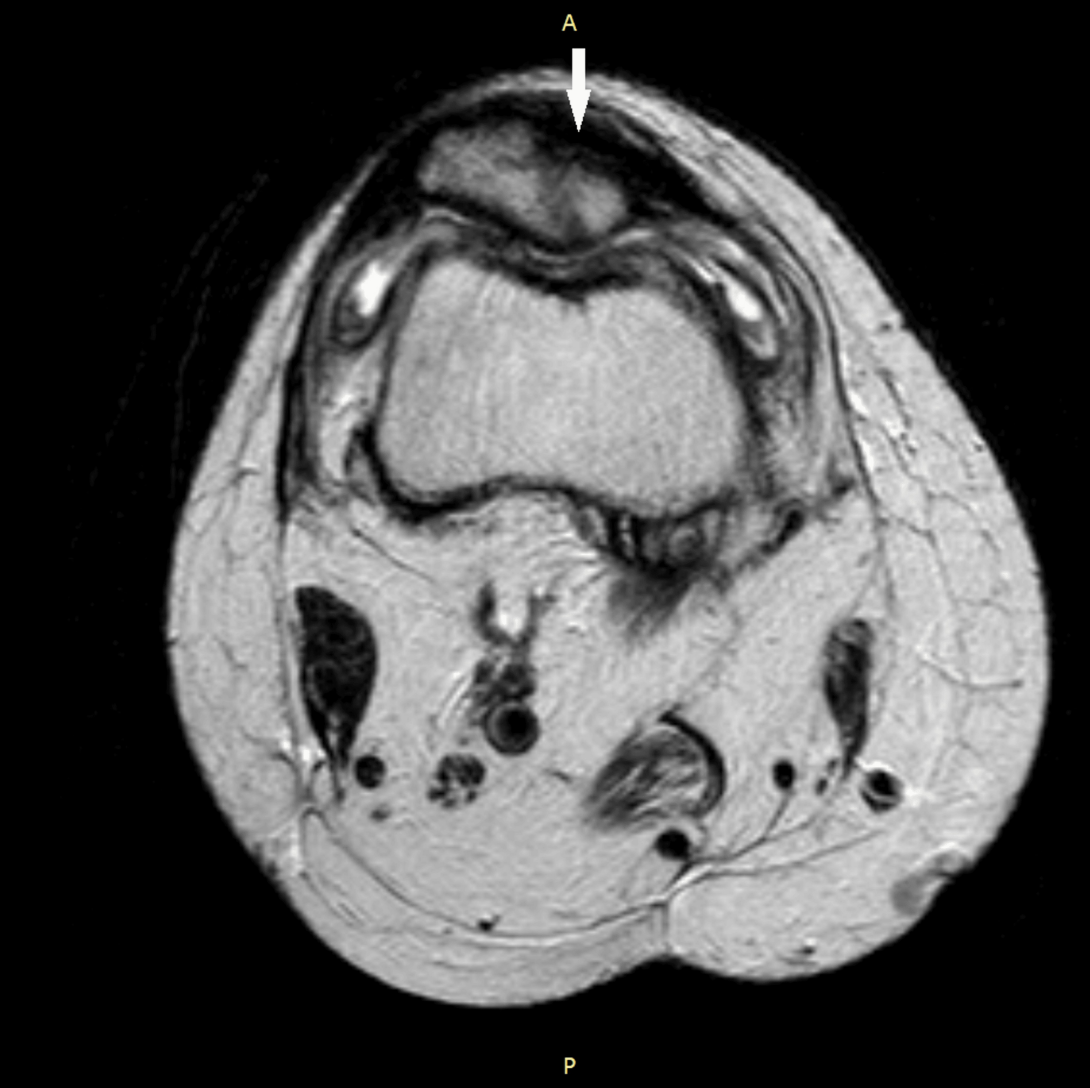
Axial view non-contrast T1-weighted MRI of the patient's right knee demonstrating a large hypodense area involving the inner substance of the patella.

**Figure 6:**
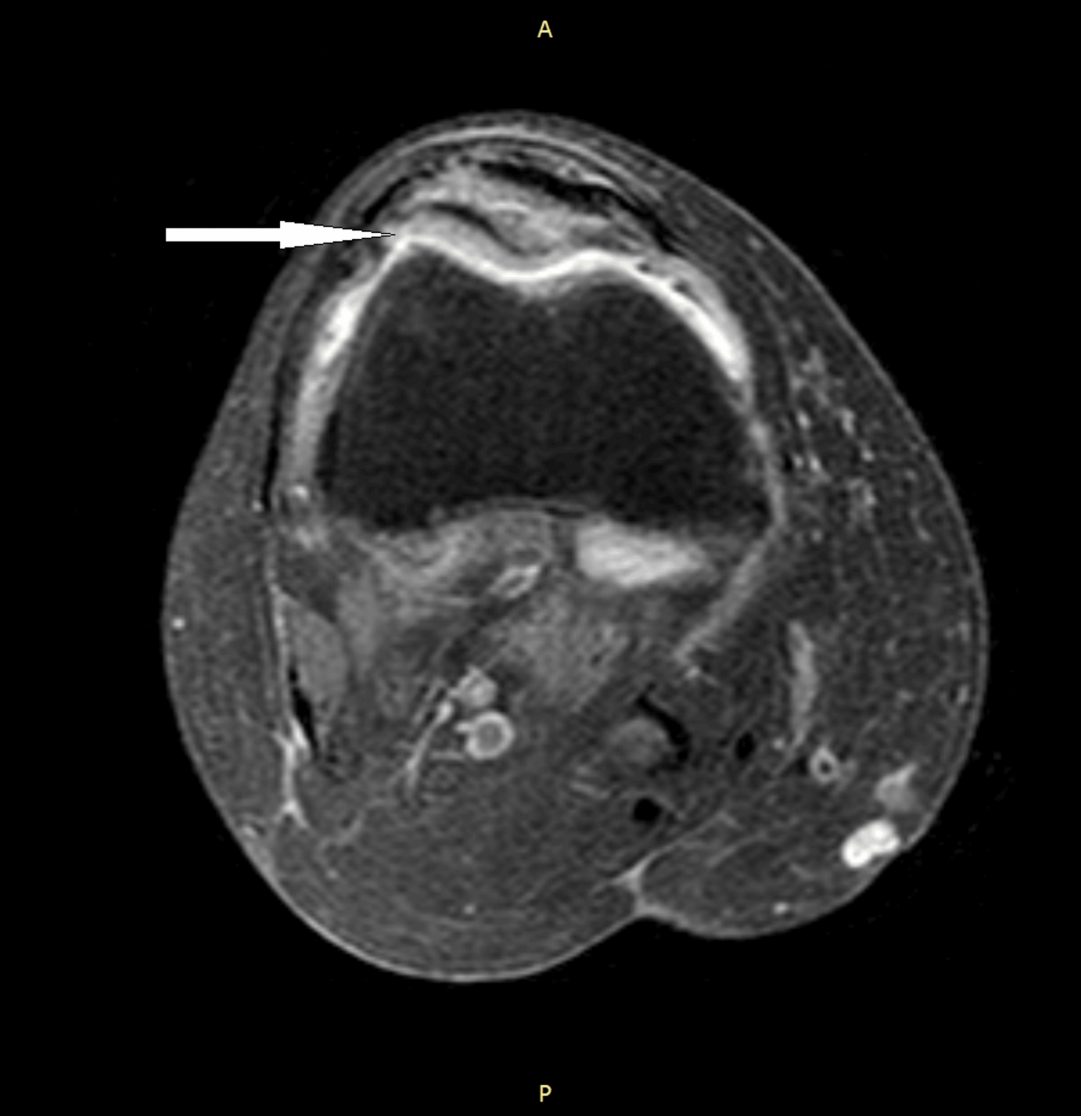
Axial view T2-weighted proton density inversion recovery MRI showing an increased signal seen within the medullary cavity of the patella demonstrating inflammation.

**Figure 7:**
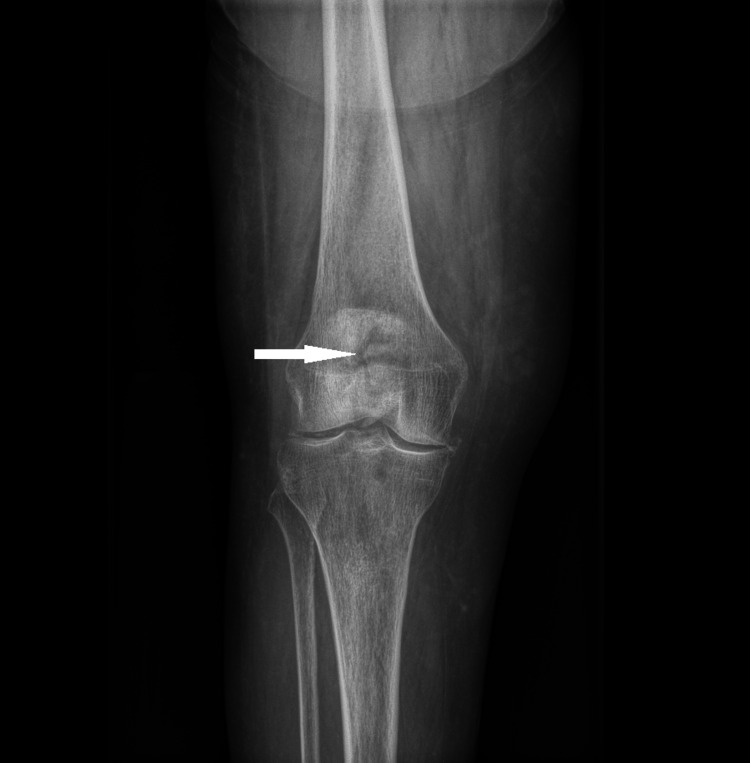
Coronal view X-ray of the patient's right knee demonstrating a comminuted fracture of the patella demonstrated by the hypodense area on the anterior aspect of the patella.

**Figure 8:**
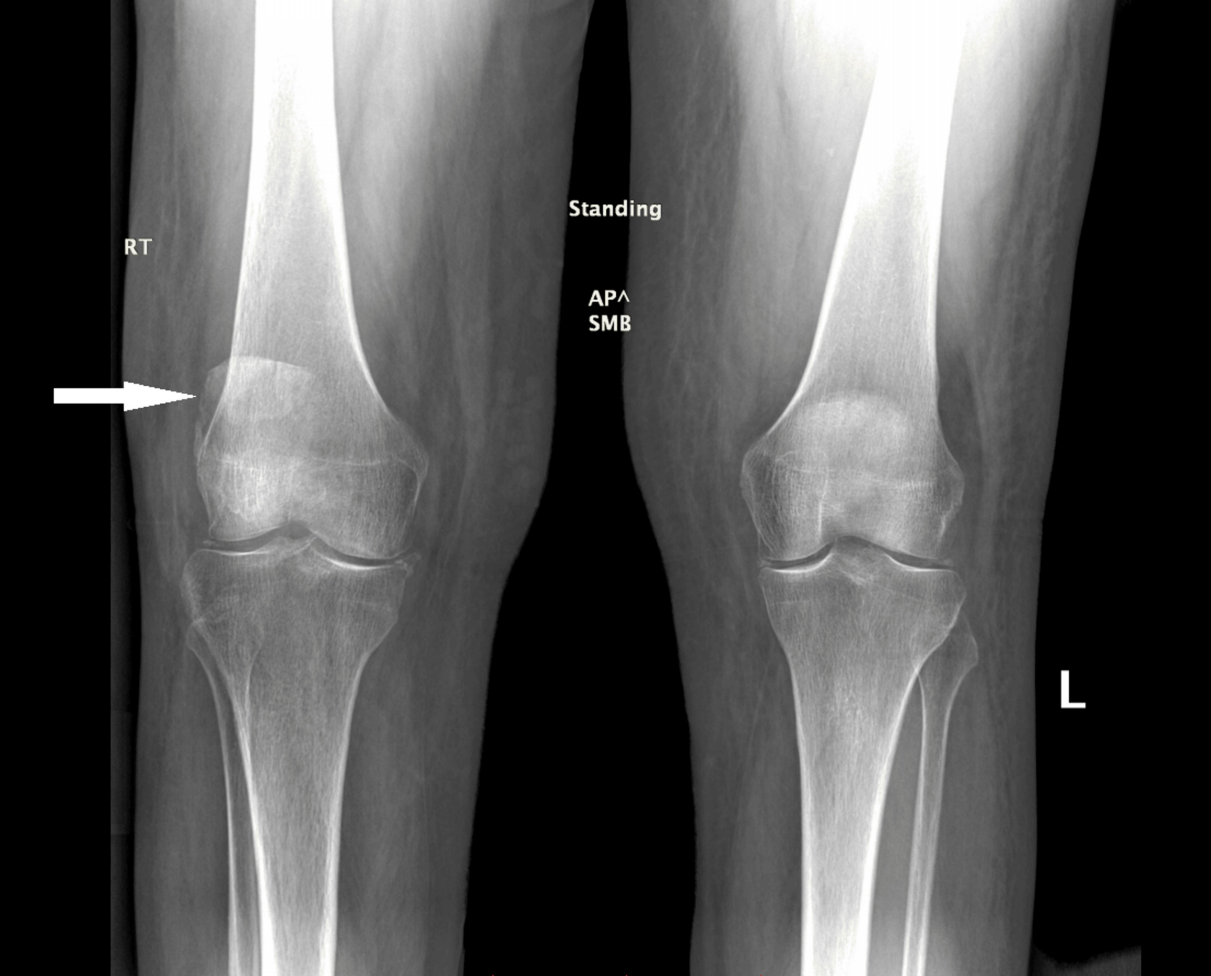
Coronal view X-ray of both of the patient's knees showing the superior displacement of patellar fragments of the right knee compared to the intact left knee.

**Figure 9:**
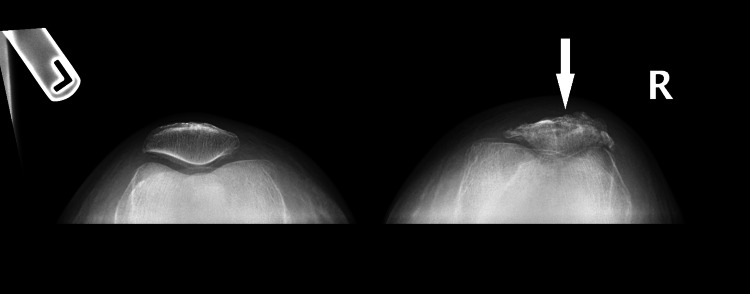
Merchant view of both of the patient's knees showing bony deformities of the right patella along with narrowing of the space between the patella and the trochlear groove.

On all imaging modalities, a comminuted fracture of the right patella can be seen (Figures 1-3 and Figures 7-9). With a lack of evidence of fusion of the fragments, the multiple MRIs demonstrate nonunion of the patient's patellar fracture (Figures 2-6). Depending on the severity, a patellar fracture can take anywhere from three to six months to heal. Signs of fracture fusion would have been seen on radiographs beginning at week 4, demonstrating the placement of fibrous tissue and cartilage [27]. However, these signs were absent on the patient's radiographs due to her osteomyelitis infection preventing the fusion of the bone. Additionally, the patient's proton density inversion recovery MRI showed increased signaling surrounding the fractured patellar fragments, indicating inflammation due to the patient's immune system fighting the infection (Figure 3 and Figure 6). It can also be concluded that this inflammation is not due to the healing process of the patella because the patient's imaging shows that there are no signs of healing in her patella.

Osteomyelitis may not have been considered initially within the differential diagnosis based on imaging alone due to the absence of osteolytic lesions observed on the patient's roentgenographic studies. Osteolytic lesions take up to two weeks in children and longer in adults to appear on X-ray [18]; paired with the difficulty of visualizing such a relatively small area, it makes diagnosing the early stages of osteomyelitis even more challenging. However, the key indication in ordering the MRI was that the patient's pain failed to improve and was progressively worsening. The combination of the patient's abnormal ESR, CRP, and elevated WBC count supports the finding of inflammation within her knee joint as seen on the T2-weighted proton density inversion recovery MRI.

With consideration of the extent of damage of the patient's patella, the absence of sequestra, and the utilization of antibiotics alone, we would classify the patient's osteomyelitis as stage 1 according to the Cierny-Mader classification system [16]. This is due to the bacteria invasion of the medullary cavity and the space between the fracture fragments (Figure 3 and Figure 4). Creating a treatment plan for the patient involved the careful consideration of her previous medical history that placed her at risk for complications such as blood clots, surgical site infection, and improper wound healing, following debridement surgery. Additionally, the infecting bacteria was never identified due to both negative blood cultures and knee aspirations. Therefore, consideration of antibiotics that cover a wide range of microbes that commonly cause osteomyelitis eventually leads to the utilization of a four-week course of doxycycline and cefdinir. 

Doxycycline is a tetracycline antibiotic that is commonly used for musculoskeletal infections secondary to its broad coverage of gram-positive bacteria, such as *S. aureus*, and gram-negative bacteria, including *Brucella*, *Borrelia*, or *Chlamydia* [28,29]. Due to the patient having negative peripheral blood cultures, utilizing doxycycline as an antibiotic would be beneficial for eliminating *S. aureus* specifically as this is the most likely pathogen to cause osteomyelitis. Additionally, doxycycline is a good agent for osteomyelitis in particular because it has bone penetration characteristics, making it more effective against infections in bones specifically [29]. Specifically, doxycycline contributed to the healing of the patient's patellar fracture by promoting bone remodeling and reducing the inflammation in the area. Doxycycline impacts bone remodeling by inhibiting matrix metalloproteinase (MMP) activity, which is crucial to the healing of acute injuries by preventing the breakdown of extracellular matrix, collagens, glycoproteins, and proteoglycans, thereby preventing the loss of bone matrix [30]. Furthermore, doxycycline is an anti-inflammatory agent as it alters the signaling cascade in the production of both cytokines and chemokines [31]. This ultimately prevents excessive inflammatory responses, allowing our patient's patellar fracture and infection to heal.

Cefdinir is a third-generation cephalosporin. Cefdinir, similar to doxycycline in the fact that it has broad-spectrum coverage of bacteria, is effective against both gram-negative and gram-positive aerobic organisms [32]. While covering these bacterial species, cefdinir is also stable against hydrolysis when in the presence of 13 of the most common beta-lactamases and is effective against skin and skin-structure infections [32]. It can be noted that a third-generation cephalosporin is able to effectively treat and eliminate the most common causes of osteomyelitis including coagulase-negative staphylococci, streptococci, and enterococci. Additionally, while the MRI demonstrated that this was a bone infection, pointing to osteomyelitis, utilizing cefdinir further would aid in the prevention of the spread of bacteria to surrounding soft tissue, due to its use in epidermal, dermal, and soft tissue infections.

## Conclusions

During the first week of starting antibiotic therapy, our patient reported that her pain was reduced to nearly zero. The patient did not have follow-up imaging performed after she completed the course of antibiotics or in the following months while her fracture was healing. She was able to achieve uninterrupted sleep and resumed walking without bracing. However, to reach a complete recovery was a long and grueling process. The patient had nine X-rays performed on her knee, with each provider stating that her fracture was healing; meanwhile, her worsening pain indicated otherwise. Baseline values such as those found in a complete blood count with differential should be ordered in patients recovering from fractures, due to their indication of infection, and could indicate a patient's risk of clot formation due to damaged blood vessels following the injury. 

Our patient made an adequate recovery utilizing antibiotics alone, which deviates from the currently established care protocols for osteomyelitis. However, these protocols are written for the treatment of long bone osteomyelitis, rather than sesamoid bones. Osteomyelitis of the long bones is often much more severe and can lead to detrimental structural instability and the spread of infection to local tissues, which makes surgical debridement necessary to remove any infected and necrotic tissue. However, since the patella is such a small area compared to long bones, it's possible that the immune and lymphatic systems can sufficiently clear infected and necrotic tissue following the elimination of the initial infection. This prevents patients from undergoing surgeries that may result in more harm than benefit due to high risks of complications. With the few reported cases of patellar osteomyelitis comes the challenge of comparing treatment outcomes with different therapies. However, we now know there is a potential to eliminate the need for surgical intervention in the patella and possibly other relatively small areas of the body.
